# Case report: Type I diastematomyelia with breast abnormalities and clubfoot

**DOI:** 10.3389/fsurg.2022.981069

**Published:** 2022-09-07

**Authors:** Shenshen Hao, Zenan Yue, Xin Yu, Zhenfu Gao, Hongke Li, Shuai Liu, Shengli Dong

**Affiliations:** ^1^ Department of Spine and Bone Oncology, General Hospital of Pingmei Shenma Medical Group, Pingdingshan, China; ^2^ Theoretical Research Office, Party School of the CPC Pingdingshan Municipal Committee, Pingdingshan, China

**Keywords:** diastematomyelia, breast abnormalities, clubfoot, surgery, case report

## Abstract

**Background:**

Diastematomyelia is a rare congenital spinal cord malformation, classified as type I or type II, with over half of the cases considered type I. However, type I diastematomyelia with breast abnormality and clubfoot is extremely rare in clinical practice.

**Case presentation:**

We admitted an 18-year-old female patient with type I diastematomyelia with breast abnormalities and clubfoot. She was underwent surgical treatment. After the surgical removal of the pressure-causing bone spur, the weakness of the right lower limb was significantly relieved. During the 22-month follow-up, there was no complication and no recurrence.

**Conclusion:**

Surgical removal of the pressure-causing bone spur can relieve symptoms in the lower limbs. However, further research is warranted to explore the breast abnormalities in patients with diastematomyelia.

## Introduction

Diastematomyelia is a rare congenital malformation of the spinal cord (1). Due to its very low incidence, no relevant epidemiological studies have been conducted. There are two types of diastematomyelia: type I, characterized by two hemispinal cords in the lesion area, each of which has its own dural sac that forms a sheath with bony or cartilaginous compartments, also known as bony diastema; and type II, characterized by a dural sac containing two hemispinal cords with a fibrous septum in the lesion area, also known as membranous diastema. Existing studies have shown that type I diastematomyelia accounts for about 61.4% of all diastematomyelia (2), and about 20% of such patients develop clubfoot (3). Type I diastematomyelia accompanied by breast abnormalities accounts for 1%–6% of all diastematomyelia, and ectopic breast mostly occurs under the axilla (4).

The pathogenesis of diastematomyelia is as yet unclear. Diastematomyelia is a kind of recessive spina bifida, which mostly occurs at the level of the middle and lower thoracic spine as well as the middle and upper lumbar spine. The medullary cone is mostly located at a lower level. Diastematomyelia is clinically rare, accounting for 4%–9% of congenital spinal deformities. It occurs mostly in childhood, and about 80% of patients have clinical signs and symptoms before the age of 5. Moreover, females are affected much more commonly than males, at an approximate ratio of 3.5:1 (5). Patients mainly develop tethered cord due to bony or fibrous intervals. Neurologic impairment is increasingly aggravated with the progression of the disease (6). Symptoms of nerve injuries in childhood mostly include unsteady walking, swaying, weakness of the lower limbs, scoliosis, and characteristic skin changes in the lower back, whereas those in adulthood include pain and long-term weakness of the affecte limbs (7).

Thus, diastematomyelia accompanied by breast abnormalities is quite rare. We could not find any report of such a case through literature review. In September 2020, we admitted a patient with type I diastematomyelia, which was accompanied by breast abnormalities and clubfoot. Based on this case, we hope to provide reference for clinical diagnosis and treatment of similar cases. Here, we try to combine our experience with a review of the literature to explore the following issues: (1) misdiagnosis and missed diagnosis of diastematomyelia; (2) the association between diastematomyelia and ectopic breast and clubfoot.

## Case presentation

**Figure 1:**
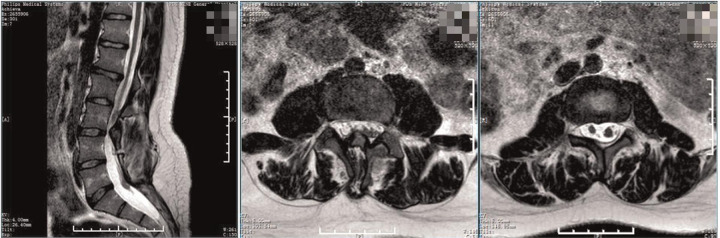
Preoperative MRI. The spinal cord was shifted down, approximately at L4 segment, with bifurcation of the lower part. The lamina of L3 segment was not fused, and the spinous process protruded into the spinal canal, resulting in spinal stenosis of the related segments.

**Figure 2:**
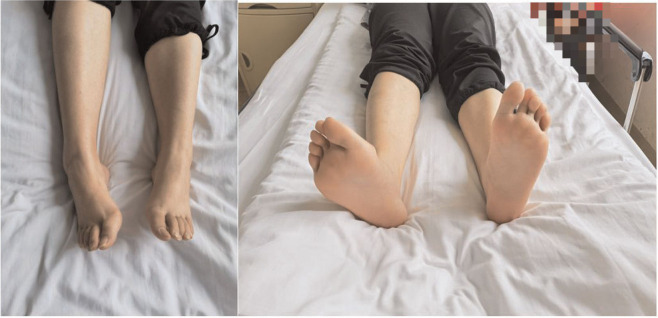
Preoperative physical examination of the lower limbs. The patient showed clubfoot of the right lower limb,while the normal of the other.

**Figure 3:**
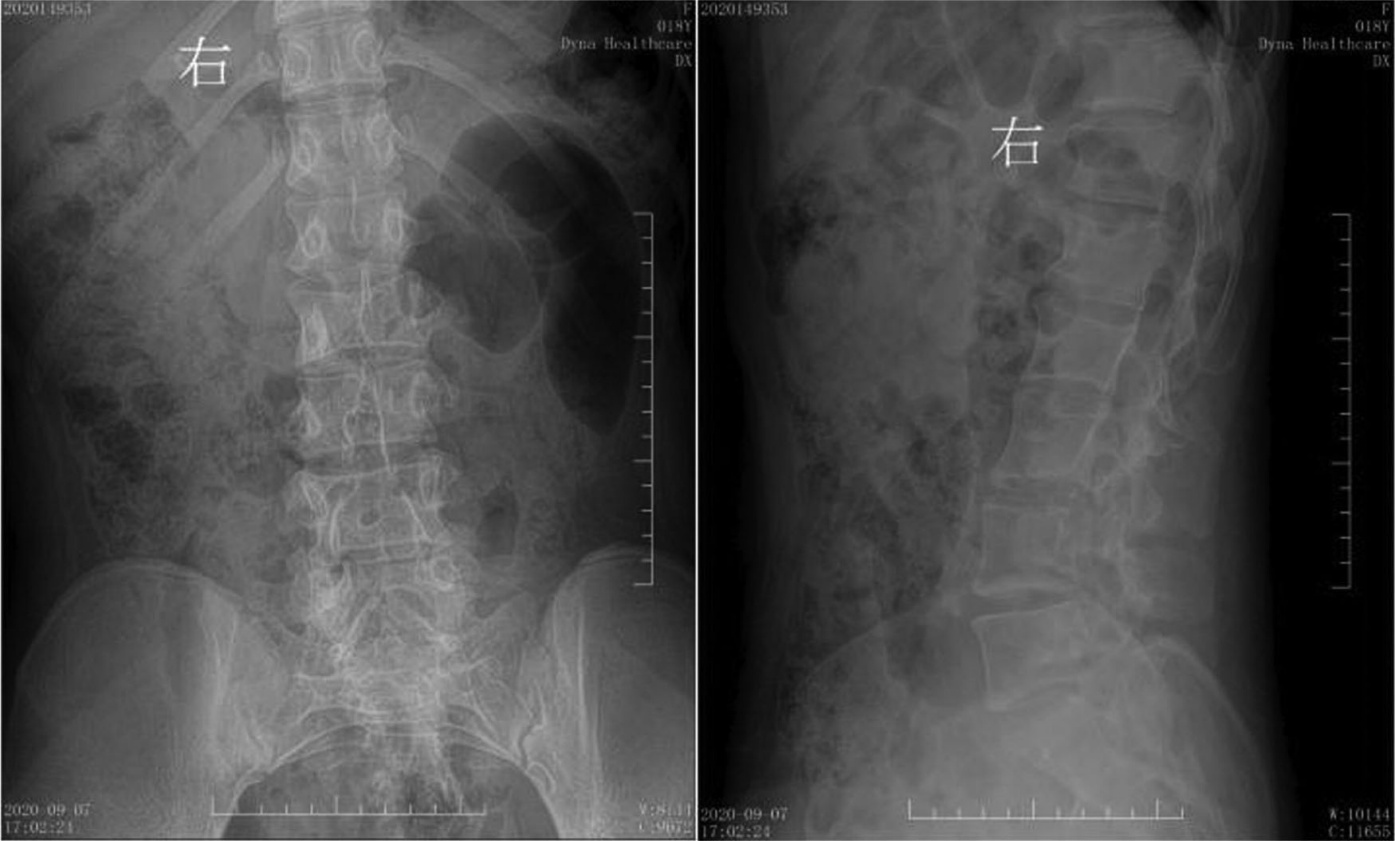
Preoperative digital radiography. Spina bifida is shown in the lumbar spine, with the L3 spinous process extending into the spinal canal.

**Figure 4:**
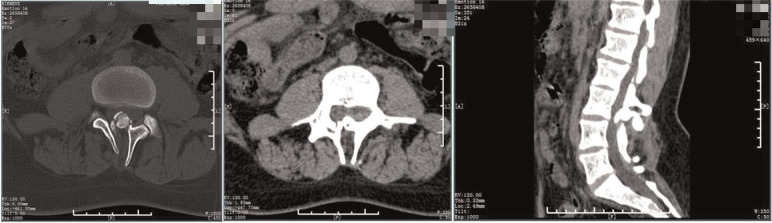
Preoperative CT. The lamina of the L3-5 vertebra were not fused, the spinous process of the L3 protrudes inward, considerating diastematorrhachia.

**Figure 5:**
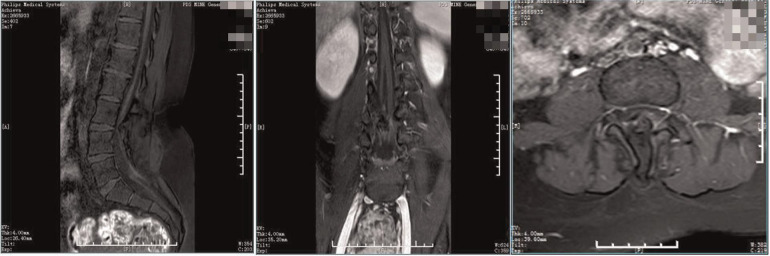
Preoperative enhanced MRI. The spine was diastematomyelia, with tethered spinal cord and terminal filament lipoma, infusion of the lamina at L3-5 segments, and local lamina thinning.

**Figure 6:**
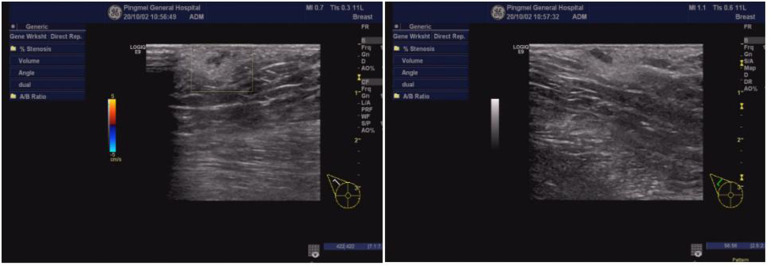
Ultrasound examination of the right armpit. An accessory breast was in the right armpit, at about 0.67  ×  0.49  ×  0.28 cm^3^.

An 18-year-old female patient was admitted to the hospital with the chief complaint of “weakness of the right lower limb that has gradually worsened for more than seven years.” The patient had right lower extremity weakness of unknown origin, which developed more than seven years ago, with slightly limited walking ability. The symptoms gradually worsened, accompanied by waist pain and thinning of the right lower extremity, leading her to seek medical attention at our hospital. Whereupon magnetic resonance imaging (MRI) indicated diastematomyelia. The spinal cord was shifted down, approximately at the level of the L4 segment, with bifurcation of the lower part, but no abnormal signals. The lamina of the L3 segment was not fused, and the spinous process protruded into the spinal canal, resulting in spinal stenosis of the related segments. There was neither obvious abnormality in the lumbar intervertebral disc, nor was there any thickening of the ligamentum flavum (Figure 1). The physical examination revealed lumbar tenderness and percussive pain, and irregular palpable spinous processes in the lower back. The right lower limb was slightly thinner than the left. The muscle force of the right and left lower limbs was graded at level 4 and 5, respectively. The patient suffered clubfoot of the right lower limb (Figure 2), but no abnormality in the left lower limb, negative findings in the Bragard sign of both lower limbs, and normal blood supply of both lower limbs. Digital radiography indicated spina bifida, with the L3 spinous process protruding into the spinal canal (Figure 3). Computed tomography (CT) indicated lamina infusion of the L3-5 segments, with inward protrusion of the L3 spinous process into the spinal canal and local spinal stenosis, indicating a congenital diastematorrhachia (Figure 4). Enhanced MRI showed diastematomyelia, with tethered spinal cord and terminal filament lipoma, infusion of the lamina at L3-5 segments, and local lamina thinning, suggesting spina bifida (Figure 5). There was a palpable “peanut grain”-like subcutaneous hard mass in the right armpit. Ultrasound examination revealed an accessory breast in the right armpit, accompanied by sheet-like hypoechoic sound, about 0.67 × 0.49 × 0.28 cm^3^ (Figure 6). Diagnoses were as follows: (1) type I diastematomyelia; (2) spinal canal stenosis of the lumbar segment (bony encroachment); (3) tethered cord; (4) clubfoot (right); (5) abnormal breast (right armpit); and (6) spina bifida (lumbar spine). After preoperative preparation, posterior lumbar spinal canal resection with spinal canal expansion and decompression and spinal nerve exploration were conducted under general anesthesia. And, the core of surgery was excising the spur.

**Figure 7:**
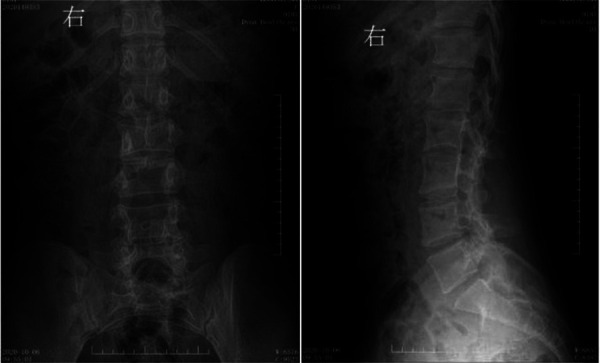
Postoperative digital radiography. The spine was bifida, with no display of some lumbar spinous processes.

**Figure 8:**
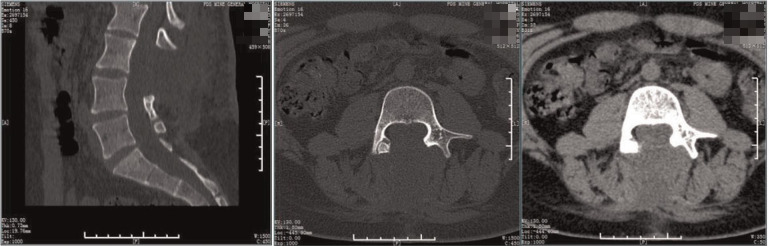
Postoperative CT. The spur was excised and the laminae of the L3-5 segments were not fused.

**Figure 9:**
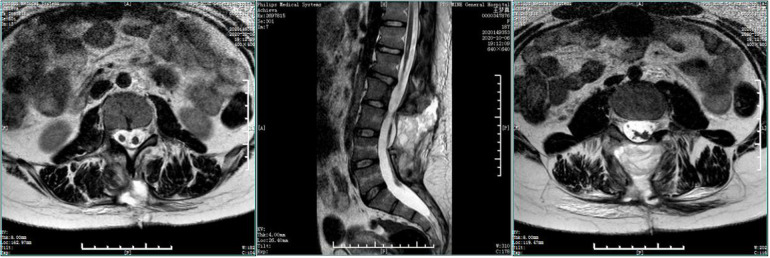
Postoperative MRI. The compression of the spinal cord was removed, with diastematomyelia and tethered cord, without fusion of the L4-5 vertebral arches.

Postoperatively, the pathological results suggested normal bone tissue,, which means that the spur was bony. Digital radiography indicated spina bifida, with no display of some spinous processes of the lumbar spine (Figure 7). CT showed that the soft tissues of the L3 segment were swollen, but the laminae of the L3-5 segments were not fused (Figure 8). The MRI indicated diastematomyelia and tethered cord with no fusion of the L4-5 vertebral arches (Figure 9). The right lower limb weakness was gradually relieved to level 5 of the muscle force, and the lumbar pain gradually disappeared. There were no evident changes in the circumference of the right lower limb and the morphology of the clubfoot within the 1-month follow-up. The patient was very satisfied with the treatment strategy we have chosen. During the 22-month follow-up, there was no complication and no recurrence.

## Discussion

Diastematomyelia is a rare congenital malformation of the spinal cord. Due to its very low incidence, no relevant epidemiological studies have been conducted, and the pathogenesis of diastematomyelia is as yet unclear. The “unified theory” proposed by Pang (8) in 1992 is currently recognized as the theory of pathogenesis, which proposes that diastematomyelia originates from a basic abnormality in the embryonic stage. Between the 3rd and 4th weeks of pregnancy, the endoderm and ectoderm adhere, which leads to cracking of the notochord. The cracked notochord causes splitting of the upper nerve plate. Meanwhile, the surrounding mesenchyme is concentrated around the nerve plate to form an internal mesenchymal tract between the cracked notochord and the nerve plate. Pluripotent mesenchyme can differentiate into fibers, cartilage, bone tissue, blood vessels, adipose tissue, and myoblasts, all of which sagittally divide the spinal cord on the midline, forming a diastematomyelia. Moreover, deformities of the spine and skin develop based on the embryological changes (6).

Diastematomyelia develops insidiously, progresses slowly, and is easily missed and misdiagnosed. About 50% of patients will have an increased risk of progressive neurological impairment with aging (9). Zhao et al. (10) believe that while neurological surgery cannot improve a patient's preoperative neurological impairment, it can prevent or block worsening of the neurological impairment. Mahapatra et al. (2) believe that the risk of neurological impairment increases with age, and that all patients should be treated with preventive surgery, even if they are asymptomatic. Therefore, surgical treatment is the only effective method for treating diastematomyelia, and a surgery should be scheduled once the diagnosis is confirmed (11). Clinicians can easily confirm diastematomyelia based on radiography, CT, and MRI. Diastematomyelia should be suspected if there is lumbar discomfort, scoliosis, and abnormalities of lower limbs.

In this study, we reported a special case of type I diastematomyelia accompanied by both ectopic breast and clubfoot. Abnormal development of the spinal cord may induce clubfoot. For example, due to the different growth speeds of the spinal cord and spine, the spinal cord may experience a traction that causes mechanical damage, further disturbing the nerve stability of the lower limbs, and eventually triggering clubfoot. Chen et al. (12) reported a case of diastematomyelia misdiagnosed as clubfoot. Therefore, we believe that for patients with clubfoot, diastematomyelia should be suspected, based on related examinations of the spinal cord, such as radiography, CT, and MRI, to limit any impact on disease diagnosis and treatment.

The normal development process of mammary glands is as follows: when the embryo develops for about 6 weeks, there are 6–8 mammary gland primordia-like paired “buds” on the ectodermal ridges on both human ventral sides. Except for one pair of mammary gland primordia on the chest that continue to develop, other paired buds in other positions gradually atrophy and disappear, eventually forming normal mammary glands. Ectopic breast can be caused when the mammary gland primordia outside the normal breast are not degenerated or degenerate incompletely. Milk lines are a pair of ventral epidermal ridge, extending from the axilla to the groin. Ectopic breasts are present in only 1%–3% of women, most of whom are of childbearing age (13). About 90% of ectopic breasts occur along the milk line during the embryonic stage, extending from the front axillary line, through the normal nipple position, to the inside of the femoral triangle. The remaining 10% of ectopic breasts occur outside the milk line (4). Similar to normal breasts, ectopic breasts can also have periodic changes due to endocrine secretion. Generally, ectopic breasts that are asymptomatic and soft-textured can be reviewed regularly without immediate surgical treatment. Due to the possibility of ectopic breast cancer, surgical resection is recommended in cases with remarkable changes in appearance, obvious induration inside the tissue, and obvious pain symptoms (14).

Diastematomyelia with breast abnormalities is quite rare in clinical practice. Through a literature review, we found only one report of diastematomyelia with breast abnormalities. Shen et al. (15) reported a 12-year-old child with diastematomyelia accompanied by breast abnormalities on the back, which was suspected of being related to the combined chromosomal malformation. The following conditions may result in diastematomyelia accompanied by ectopic breast: both diastematomyelia and ectopic breast are triggered by incorrect expression during embryogenesis, and there is a certain overlap in the appearance time of them. So, some unknown factors that occur during embryogenesis induce the concurrence of diastematomyelia and ectopic breast. However, this is only an author's supposition, and further research on the specific etiology and mechanism is still required.

## Conclusion

The occurrence of diastematomyelia is a complicated process resulting from the interaction of environmental factors and genetics. Diastematomyelia is also triggered by the incorrect expression of many different genes, and the specific mechanism remains unknown. As such, the limitation of our study is that we were unable to find relevant research reports on the mechanism and causes of diastematomyelia with ectopic breast and clubfoot from the existing literature. Therefore, further research on this very rare and complex disease is needed in the aspects of genetics and embryology. Our case report, showing astisfactory surgical results, on this very rare and special case of diastematomyelia with both ectopic breast and clubfoot will provide reference for future relevant research.

## Data Availability

The original contributions presented in the study are included in the article/Supplementary Material, further inquiries can be directed to the corresponding author/s.
